# Role of the complement system in normocomplementemic glomerulopathies

**DOI:** 10.1590/1414-431X2026e15624

**Published:** 2026-11-09

**Authors:** K.Z.C. Eid, R. Zen, P.F. Abreu, C.B. Dias

**Affiliations:** 1 Divisão de Nefrologia, Faculdade de Medicina, Universidade de São Paulo, São Paulo, SP, Brasil; 2 Disciplina de Nefrologia, Departamento de Medicina, Universidade Federal de São Paulo, São Paulo, SP, Brasil

**Keywords:** Kidney diseases, Glomerulonephritis, Complement system proteins, Immunoglobulin A, Glomerulonephritis, Membranous, Anti-neutrophil cytoplasmic antibody-associated vasculitis

## Abstract

Complement pathway activation is part of the innate and adaptive immune responses, playing an important role in the defense against microorganisms that cause infection. That activation occurs when a set of proteins and other molecules interact, triggering reactions that are amplified in order to form the membrane attack complex, which is responsible for attacking pathogens. The complement system plays a complex role in glomerulopathies, contributing to tissue inflammation and glomerular injury. In some glomerular diseases, the participation of complement system pathways is confirmed by decreased serum concentrations of specific complements, mainly C3 and C4. In other glomerular diseases, these serum concentrations remain normal, and there is a debate about the importance of complement activation in tissue for the maintenance and worsening of glomerular injury. Such diseases include immunoglobulin A nephropathy, membranous nephropathy, and antineutrophil cytoplasmic antibody-associated vasculitis. Understanding the role of the complement system in the pathophysiology of glomerulopathies is necessary for the development of novel therapies. Therefore, the objective of this review was to evaluate the role of the complement system in these so-called normocomplementemic glomerulopathies.

## Introduction

In some glomerular diseases, the participation of the complement system pathways is evidenced by decreases in the serum concentrations of specific complements, mainly C3 and C4. In others, where those serum concentrations are normal, there is debate about the importance of complement activation in the maintenance and worsening of glomerular injury. Such diseases include immunoglobulin A (IgA) nephropathy, membranous nephropathy, and antineutrophil cytoplasmic antibody (ANCA)-associated vasculitis (AAV) (1). Understanding the role of the complement system in the pathophysiology of glomerulopathies is necessary for the development of novel therapies. Therefore, the objective of this review was to evaluate the role of the complement system in these so-called normocomplementemic glomerulopathies.

## The complement system

The complement system is one of the main effector mechanisms of adaptive (acquired) immunity and an important effector mechanism of innate immunity. It constitutes a set of plasma and cell surface proteins that interact with each other and with other molecules of the immune system in a coordinated manner. These proteins function to opsonize microorganisms and promote the recruitment of phagocytes to the site of infection, thereby initiating and modulating the immune response. The proteins that compose the complement system are naturally inactive and become activated in specific situations, generating products that participate in various effector functions of the system (2).

Complement system activation depends on a series of intracellular signals and involves proteolytic cascades, in which enzymes are modified and become active proteases with cleavage ability. These proteases generate effector products of the complement system, thus creating a system that sequentially amplifies its response (2). This system is activated by three different pathways, as described below.

### Classical pathway

The classical pathway, the first to be discovered, begins with C1q, which recognizes antibodies bound to antigens through the Fc regions of the antibodies. These antigen-antibody complexes can be soluble, bound to cell surfaces, or bound to the extracellular matrix. Each C1q molecule must bind to at least two immunoglobulin heavy chains to be activated. Such immunoglobulins include certain IgG types and IgM. Among the IgGs, those that bind to C1q and activate the classical pathway are IgG1 and IgG3. Each Fc region of an immunoglobulin has only one binding site for C1q. Subsequent to that binding, there is formation of the C1 complex (C1q plus two C1s proteases located in the center and one C1r protease on the outside of each C1s molecule), which cleaves C4 into C4a and C4b and then cleaves C2 into C2b and C2a. Their binding results in the formation of C4bC2a (C3 convertase), the function of which is to cleave C3 into C3a and C3b (2). The classical pathway is one of the main effector mechanisms of the humoral component of the adaptive immune response. When C1q becomes activated, it activates C4, its natural substrate. Although the C4 cleavage product C4d has no biological function, it is considered a residual tissue marker and its presence is generally interpreted as a sign of classical pathway activation; however, C4d can also be generated from the lectin pathway (3).

### Alternative pathway

In the alternative pathway, complement activation occurs spontaneously and continuously at a low rate (referred to as the tick-over mechanism). The mechanism involves C3 combining with a water molecule, thus forming C3(H_2_O), which recruits complement factors B and D, amplifiers of this pathway. Once the C3b formed has bound to the surface of a pathogen, it can function as an opsonin, marking pathogens as foreign cells and stimulating phagocytosis by the host. In addition, it can propagate the activation of the complement system in an amplification loop by binding to factor B and undergoing cleavage by factor D to create the C3bBb complex, another C3 convertase that is stabilized by the P factor (properdin), to cleave new C3 molecules into C3a and C3b. Finally, C3b can bind to C3bBb or C4bC2a to form C5 convertase, which cleaves C5 into C5a and C5b, ultimately leading to the formation of the membrane attack complex (MAC) (2,4). Properdin, also called factor P, is produced by leukocytes and is an enzyme whose main function is to bind directly to microbial targets to provide a platform for the assembly of C3 convertase, increasing the stability of this complex (4,5).

### Lectin pathway

The lectin pathway is triggered by the binding of pattern recognition molecules to specific structures of pathogens or altered autoantigens. Mannose-binding lectin (MBL) is the main recognizer of mannose residues in bacterial glycoproteins and glycopeptides. It binds to the MBL-associated serine proteases (MASP). These proteases, which are inactive proteolytic enzymes with function and structure similar to those of C1r and C1s, are designated MASP1, MASP2, and MASP3 (2). Each MASP has a different effect on the lectin pathway (4): MASP-1 activates MASP-2 and can cleave C2 but not C4; MASP-2 cleaves and activates C4 and C2 to form C3 convertase; and MASP-3 does not cleave C4 or C2 but is responsible for activating factor D, which is key to the activation of the alternative pathway.

All three complement pathways share the formation of C3 convertase, which, when bound to other complement proteins, forms C5 convertase, a protease that cleaves C5 into C5a and C5b. Remaining bound to the surface of microbial cells, C5b initiates the formation of a conglomerate of the complement proteins C6, C7, C8, and C9, which form the MAC, a multimeric structure that forms pores in the membranes of target cells, causing the lysis of cells in which complement has been activated. The anaphylatoxin C5a contributes to a thrombotic process by inducing platelet aggregation (4). It also plays an important role in AAV. Activation of the complement system, by any of the initial pathways, is rapidly amplified, mainly through C3b, which generates even more C3b and thousands of other molecules that can form and deposit on cell surfaces (2). Figure 1 illustrates the integration between the pathways and factors D, B, and MASP-3.

**Figure 1:**
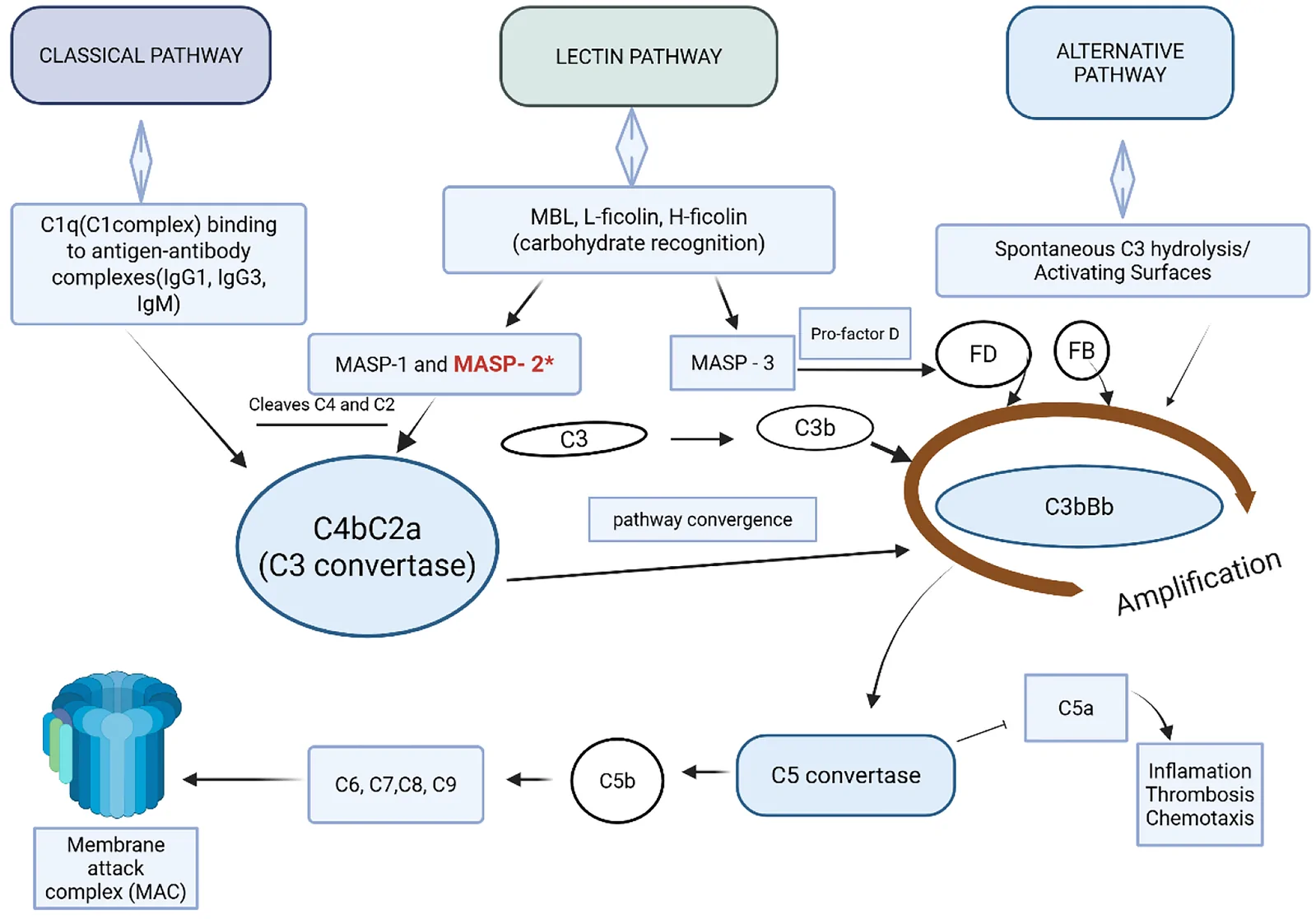
Integration of the classical, lectin, and alternative complement pathways. The classical pathway is initiated by C1q (C1 complex) binding to immune complexes containing mainly IgG1, IgG3, or IgM. The lectin pathway is triggered by carbohydrate recognition by mannose-binding lectin (MBL) and ficolins and proceeds through MASP-1 and MASP-2 to cleave C4 and C2, generating the shared C3 convertase (C4bC2a); MASP-2 is the main enzyme responsible for this cleavage. The alternative pathway can be initiated by spontaneous C3 hydrolysis and functions as the central amplification loop via the C3bBb C3 convertase, dependent on factors B and D; MASP-3 contributes by activating pro-factor D. Progression to the terminal pathway occurs after formation of the C5 convertase, which derives from C3 convertases by addition of C3b (C4bC2aC3b or C3bBbC3b), culminating in generation of C5a and C5b and assembly of the membrane attack complex (MAC, C5b-9).

## The role of C3 and the amplification and stabilization pathways of the complement system

Because C3 plays a crucial role in the continuous activation and cleavage of this system, in addition to its participation in some glomerular diseases, it is necessary to better understand how it functions. In plasma, C3 is continuously cleaved at low rates in a process called “tickover” of C3 (6).

The C3 molecule contains a thioester domain, and when it is cleaved into its largest fragment, C3b, it undergoes a conformational change that exposes this domain. This exposure allows the C3b molecule to bind to cell surfaces by forming ester bonds. If these bonds do not form, C3b remains in the so-called fluid phase, and the thioester binding site is hydrolyzed, which prevents amplification of the cascade. The “post-cleavage” conformational change in C3b also exposes a binding site for a plasma protein called Factor B, which binds to C3b on the surface of microbial or host cells, forming C3bB. Factor B is then cleaved by another protease, Factor D, producing two fragments: a larger one, Bb, which remains bound to C3b, and a smaller one, Ba. The complex formed by C3b and Bb is called C3bBb and functions as the C3 convertase of the alternative pathway, cleaving more C3 and amplifying the cascade.

Complement factor D is synthesized in the liver, immune cells, and macrophages, being stored in and secreted into the bloodstream by adipocytes. It is filtered in the glomeruli and completely reabsorbed by the tubules, with levels of this factor expected to be higher in patients with lower glomerular filtration rate (GFR), although it is still unknown whether this can worsen kidney disease (4). It plays an essentially catalytic role in the formation of C3 convertase and in the activation and function of the alternative complement pathway. Factor D is required not only in the initial step of the pathway but also for the propagation of the complement system through amplification. Additionally, because the classical and lectin pathways also use the amplification loop of the alternative pathway, factor D affects the outcomes of these pathways. Since factor D is the rate-limiting component of the alternative pathway, its concentration ultimately controls the activity and output of that pathway, including amplification. Thus, the alternative pathway - mediated by C3 production, opsonization, and cell lysis - is limited by factor D (4).

Regulatory proteins found only in normal host cells inhibit the activation of the complement system. These proteins are an adaptation of normal cells and function to minimize the damage that complement causes to host cells (2). Microorganisms lack these regulatory proteins, which allows complement activation to occur on their surfaces. Complement factor H is abundant in plasma, with a normal serum concentration of approximately 400 mg/L, regulating the alternative complement pathway in both the fluid phase and on cell surfaces. It is produced in the liver, as well as in endothelial cells and the renal mesangium. Factor H impedes the binding of Bb to C3b, accelerates the decay of C3 convertase, and acts as a cofactor for the proteolytic inactivation of C3b mediated by factor I (7). Mutations, autoantibodies against factor H, and factor H deficiency have been associated with diseases such as hemolytic uremic syndrome and C3 glomerulopathy (1).

Understanding of the complement system’s role in glomerular diseases has advanced considerably in recent years, including knowledge of classical pathway activation in lupus nephritis and dysregulation of the alternative pathway in C3 glomerulonephritis. The formation of immune complexes triggers activation of the classical pathway in some diseases, while in others it leads to dysregulation or amplified activation of the alternative pathway. Despite different activation pathways, glomeruli are susceptible to complement system activation and activity, resulting in pathological conditions (1,6).

Kidney injury caused by complement system activation results from direct effects on renal cells through interactions with cells of the innate and adaptive immune systems. Mesangial, endothelial, and tubular epithelial cells express surface receptors for C3a and C5a. Their binding triggers an inflammatory response, which promotes vessel rearrangement and attracts immune cells through chemotaxis. This injury is mediated by C3, which induces mesangial cell proliferation and secretion of extracellular matrix, as well as stimulating tubular epithelial cells to produce proinflammatory chemokines (6).

## Markers of complement system activation glomerular diseases

### Tissue markers

Activation of the complement system generates various soluble products and protein fragments that bind to tissues and can be identified in renal biopsies, serving as markers of tissue inflammation. Biopsies are usually immunostained for deposits of C4 and C4 fragments. After C4 is inactivated, the C4d fragment remains attached to cell surfaces. Detection of C4d is now routinely used as a marker of humoral rejection after renal transplantation and as an indicator of active disease in immune complex-mediated glomerulonephritis. In the absence of C1q, detection of C4d provides immunohistological evidence of lectin pathway activation; in the presence of C1q, it indicates activation of the classical pathway. In the absence of C1q or C4d, detection of C3 indicates an alternative pathway-mediated process (8). Another marker studied in renal tissue, mainly in IgA nephropathy, is properdin. Detection of properdin in IgA nephropathy indicates that, in addition to the lectin pathway, the alternative pathway can be activated in this disease (9).

### Serum and urinary markers

Low serum levels of C3 and C4 are generally associated with inflammatory activity in patients with autoimmune diseases. The serum levels of these proteins are determined by their synthesis in the liver and by their consumption in pathological conditions. Therefore, their levels can be altered under specific conditions, such as being reduced in patients with cirrhosis. However, even in diseases involving the complement system, serum levels might remain normal because local activation can cause tissue injury without depleting the plasma pool of C3 or C4, or because complement activation can occur from locally produced C3 (10).

In addition to C3 and C4, other serum markers indicate the involvement of the complement system in the pathogenesis of some glomerular diseases, such as Factor H, Factor D, and properdin. The normal plasma level of properdin (4-6 µg/mL) is low compared with those of other components of the alternative complement pathway (11). Changes in its concentration could impact local activation levels of the alternative pathway; therefore, it has been proposed that properdin release by neutrophils is a major determinant of alternative pathway activation (12).

Urinary markers have also been identified as indicators of complement activation. Brenchley et al. (13) showed that urinary C3d and C5b-9 are biomarkers of glomerular injury in patients with membranous nephropathy. A recent study demonstrated that, in patients with IgA nephropathy, high levels of urinary C4d are strongly associated with severe glomerular damage, increased proteinuria, hematuria, and renal dysfunction (14). An important advantage of these urinary markers is the ease of serial collection for comparative assessment of the progression of these glomerular diseases and possibly the response to treatment.

Table 1 summarizes some serum, urinary, and tissue biomarkers studied in understanding the pathogenesis of the complement system and glomerular diseases (1,3,5,8,10,11,13-21).

**Table 1:** Tissue, serum, and urinary biomarkers of complement activation in glomerular diseases. The table summarizes commonly used clinical and research biomarkers to infer complement activation in kidney disease, indicating the pathway or cascade step suggested, the usual detection method, and key interpretive caveats. In general, C4d reflects C4-dependent activation (classical or lectin pathway) and should be interpreted together with C1q and the immunoglobulin pattern; alternative pathway axis markers (for example, Bb, properdin, and C3 deposition) reflect C3 amplification; and terminal pathway markers (C5b-9) indicate downstream effector activation. Urinary biomarkers may enable serial monitoring and suggest intrarenal activation but should be interpreted in the clinical and histopathologic context.

Compartment	Marker	What it indicates (pathway/step)	Usual method	Practical interpretation
Tissue	C4d (glomerular deposition)	C4 cleavage-dependent activation (classical or lectin pathway)	IF/IHC	Does not distinguish classical *vs* lectin pathway on its own. Interpret together with C1q and the immunoglobulin pattern 3,15.
Tissue	C1q	Classical pathway initiation (immune complex-associated)	IF/IHC	When present with immunoglobulins and C4d, favors the classical pathway. It may be absent despite immune complexes depending on burden, technique, and lesion stage 16,17.
Tissue	C3 (and fragments)	Convergence and amplification (alternative pathway loop often dominates the final pattern)	IF	Sensitive but not specific. C3 deposits may reflect secondary amplification even when the initiating trigger was classical or lectin 16,18.
Tissue	Bb (factor B fragment) or properdin (when available)	Alternative pathway activation/amplification (formation/stabilization of C3bBb)	IHC/IF	Supports involvement of the alternative axis, especially when C3 is prominent with little evidence of C4-dependent activation 11,19.
Serum	C3 and C4	Systemic pool (synthesis *vs* consumption)	Nephelometry/turbidimetry	May be normal despite relevant renal activation. Low levels may indicate systemic complement consumption, but normal concentrations do not exclude complement involvement in renal disease 5,10.
Serum	CH50 and AH50 (when available)	Global function of classical/terminal (CH50) and alternative/terminal (AH50) pathways	Hemolytic assays	May help identify significant consumption or functional deficiency, but do not localize the site of activation (systemic *vs* local) 5,10.
Serum	sC5b-9 (soluble MAC), Bb/Ba (when available)	Terminal activation (sC5b-9) and alternative pathway activation (Bb/Ba)	ELISA	More direct markers of activation at specific steps. They are influenced by systemic inflammation and pre-analytical variables; interpret in the clinical context 1,20.
Urine	Urinary C4d (creatinine-normalized)	C4-dependent activation in the kidney (often interpreted as lectin pathway in some contexts)	ELISA	Potential noninvasive biomarker for risk stratification and serial monitoring. Interpret alongside proteinuria, hematuria, and kidney function 14,21.
Urine	Urinary C3dg/C3d	Upstream activation and C3 breakdown in the urinary compartment	ELISA	May reflect local activation and/or glomerular passage with tubular processing. Influenced by proteinuria and tubular integrity; best interpreted as serial trends rather than a single value 13,21.
Urine	Urinary C5b-9	Terminal pathway activation in the kidney	ELISA	May indicate active complement-mediated injury in selected settings. Varies with proteinuria, urine flow, and sample handling; useful for serial follow-up 13,21.
Urine	Urinary factor H (when available)	Engagement/regulation associated with the alternative pathway in the kidney	ELISA	May complement urinary activation markers and suggest alternative pathway involvement but does not replace clinical and histologic assessment 8,20.

## Membranous nephropathy and the complement system

Primary membranous nephropathy is one of the most common causes of nephrotic syndrome in adults worldwide. It is characterized by granular deposits of IgG and C3 in peripheral capillary loops, together with thickening of and spicules in the glomerular basement membrane. Studies of this disease began with the Heymann model in rats, which demonstrated that IgG antibodies formed subepithelial immune deposits through binding to a rat podocyte protein, megalin, and that proteinuria was mediated by the C5b9 complex attacking the podocyte (22). The C5b9 attack activates several signaling pathways that ultimately promote extracellular matrix production, thickening of the glomerular basement membrane, and the development of spicules. In addition, C5b9 contributes to alterations in podocyte DNA, interrupting the cell cycle, leading to podocyte loss in the urine and the development of sclerosis. In experimental models of membranous nephropathy, with depletion or genetic deficiency of complement cascade elements, complement-mediated injury has been shown to be mainly due to formation of the MAC in podocyte cells (23).

In humans, membranous nephropathy is mediated by antibodies, mostly IgG4, against the M-type phospholipase A2 receptor (PLA2R), a transmembrane glycoprotein present in podocytes. Positivity for anti-PLA2R antibodies is seen in 70-85% of membranous nephropathy cases (24,25). In addition to its specificity for diagnosis, such positivity also correlates with disease activity, treatment response, and risk of recurrence in transplant recipients. Antibodies against other podocyte proteins have been described in anti-PLA2R-negative membranous nephropathy, such as antibodies against thrombospondin type 1 domain-containing 7A, reportedly seen in 3% of cases, as well as other recently discovered antibodies. Recognition of membranous nephropathy as an autoimmune disease has changed its diagnosis and therapeutic approach (25,26).

Although IgG4 does not bind to C1q and therefore does not activate the classical complement pathway, C4d deposition has been detected in at least 84% of patients with membranous nephropathy (27). Detection of C4d on immunohistochemistry, as previously stated, indicates activation of the classical or lectin pathway. However, observations that MBL and galactose-deficient IgG (IgG4) can be detected in the subepithelial space in membranous nephropathy, as well as that IgG4 can bind to MBL and activate complement via the lectin pathway (15), suggest that the pathogenesis of membranous nephropathy involves activation of the lectin pathway (Figure 2).

**Figure 2:**
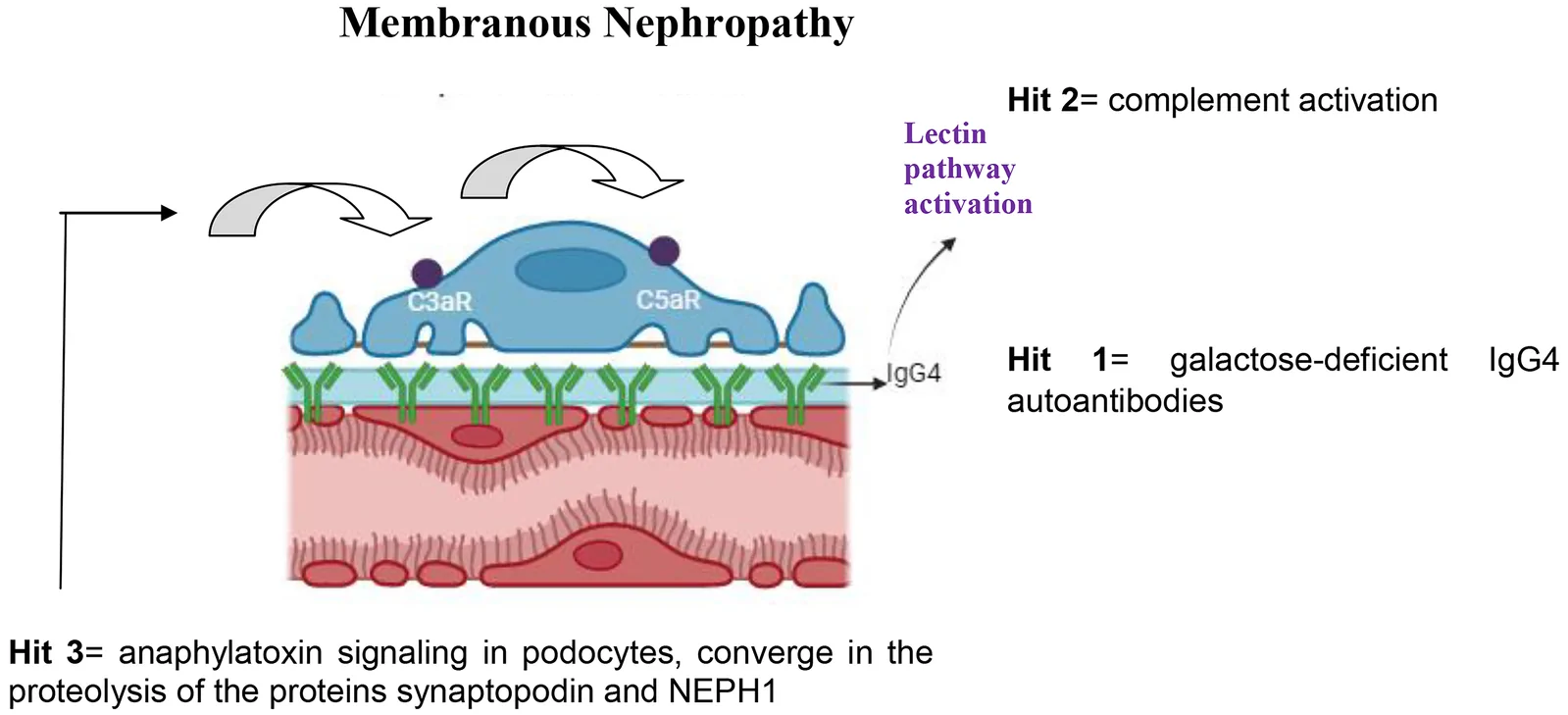
Complement system activation in membranous nephropathy. Ig: immunoglobulin.

In a recent study, Haddad et al. (28) established an *in vitro* model based on human podocytes expressing PLA2R and patient serum with IgG4. Using this model, they proposed an explanation for the pathogenesis of membranous nephropathy based on three “hits” that contribute to podocyte injury: the development of IgG4 autoantibodies against PLA2R; an altered glycosylation pattern of immunoglobulins, producing IgG4 autoantibodies capable of activating complement through the lectin pathway; and upregulation of the complement receptors C3aR1 and C5aR1, which facilitates anaphylatoxin signaling in podocytes.

The formation of the MAC and C3aR1 or C5aR1 signaling converge in the proteolysis of the proteins synaptopodin and NEPH1, which are crucial for maintaining the actin cytoskeleton and slit diaphragm. Haddad et al. (28) observed the participation of the complement system and the effector pathways that lead to podocyte injury, demonstrating that IgG4 antibodies against PLA2R activate the complement system after binding to podocytes, and that this effect is facilitated by aberrant glycosylation that allows IgG4-induced activation of the lectin pathway.

In a recent study, Ravindran et al. (29) analyzed renal biopsies from patients with membranous nephropathy, which was associated with PLA2R in seven and with EXT1/EXT2 in 21, using microdissection and mass spectrometry to identify the spectrum of complement proteins. The authors found that the most abundant complement protein in patients with anti-PLA2R-associated membranous nephropathy was C3, followed by C4, with C1 being the least common. Among the complement-regulating proteins, the most common were Factor H and its derivatives. Properdin was found at low levels, suggesting that the alternative complement pathway was not activated.

Other studies evaluating anti-PLA2R-positive membranous nephropathy found elevated urinary MAC compared with controls and concluded that complement activation was intrarenal, given that these molecules are too large to be filtered. In addition, the detection of urinary MBL and C4d correlates with anti-PLA2R-positive patients, identifying the lectin pathway as a major pathway of complement system activation but raising questions about the participation of the alternative pathway in all cases or in specific cases (21).

## IgA nephropathy and the complement system

The most common primary glomerulopathy worldwide is IgA nephropathy. It has a higher incidence in Asian populations and presents with a wide spectrum of clinical and biochemical features, ranging from asymptomatic forms to progressive loss of renal function. Up to 40% of patients progress to stage 5 chronic kidney disease within the first 10-20 years after diagnosis (30). IgA nephropathy is mainly characterized by mesangial hypercellularity, accompanied by diffuse mesangial deposits of IgA and often C3, which can also contain IgG. The pathogenesis of IgA nephropathy involves a “multi-hit” process, including the circulation of IgA1 with deficient glycosylation, specifically galactose-deficient O-glycans in the hinge region (Gd-IgA1).

Patients with IgA nephropathy have high circulating levels of Gd-IgA1, which correlate with disease severity. Gd-IgA1 exposes terminal residues of N-acetylgalactosamine, triggering the formation of autoantibodies against this defective IgA (30). This leads to the formation of immune complexes composed of hypogalactosylated IgA and an IgG or IgA antibody, or the binding of this defective IgA with a soluble factor. These immune complexes are deposited in the mesangium and activate several pathways responsible for the clinical and histological changes observed in patients (31). This pathophysiological process results from a combination of genetic and environmental factors that modulate mucosal function and trigger altered glycosylation of IgA, culminating in the injury responsible for IgA nephropathy. Recent experimental and genetic studies have provided insight into the pathogenic process that initiates the disease, including the important roles of the mucosa and immune system in activating the complement system. The classic manifestation, hematuria, which generally occurs concurrently with an infectious or inflammatory process in the airways or intestine, is possibly related to an aberrant immune response to disturbances in the mucosa (31).

Glycosylation-defective IgA1 exhibits altered biological properties compared with normal IgA1, including a greater tendency to self-aggregate (polymerize), exposure of MBL binding sites leading to complement activation, and binding to other molecules such as fibronectin, IgG, and type IV collagen (32).

The absence of C1q in biopsies of patients with IgA nephropathy indicates that the classical pathway is not the main component activated in such patients. In contrast, detection of C4d in the absence of C1q indicates involvement of the lectin pathway. Polymerized IgA can activate the lectin pathway, while monomeric IgA or immune complex formation might activate the alternative pathway, contributing to local inflammation, cell proliferation, and fibrosis (21). Colocalization of MBL with C3 and C4d in mesangial deposits has been observed in up to 25% of patients, suggesting activation of the lectin pathway (Figure 3). In some cohorts of patients with IgA nephropathy, those with C4d positivity have been found to have greater proteinuria and lower GFR at diagnosis (33-35). In a study conducted in North Korea, C3 deposition in the mesangium was associated with worse renal outcomes among patients with IgA nephropathy (18). The idea that these two complement pathways are activated simultaneously in patients with IgA nephropathy was confirmed by a recent study that found 27.3% positivity for C3bBb in renal biopsy (indicating the alternative pathway), 39.4% for C4b2a (lectin pathway in this case), and 39.4% positivity for both pathways. Another very interesting finding in this study was the correlation between activation of the alternative pathway and endocapillary proliferation and glomerular sclerosis (MEST E1 and S1, respectively), as well as a higher frequency of crescents, vascular lesions, and poorer renal survival (36). This suggests that patients with alternative pathway activation in their pathogenesis are more severely affected.

**Figure 3:**
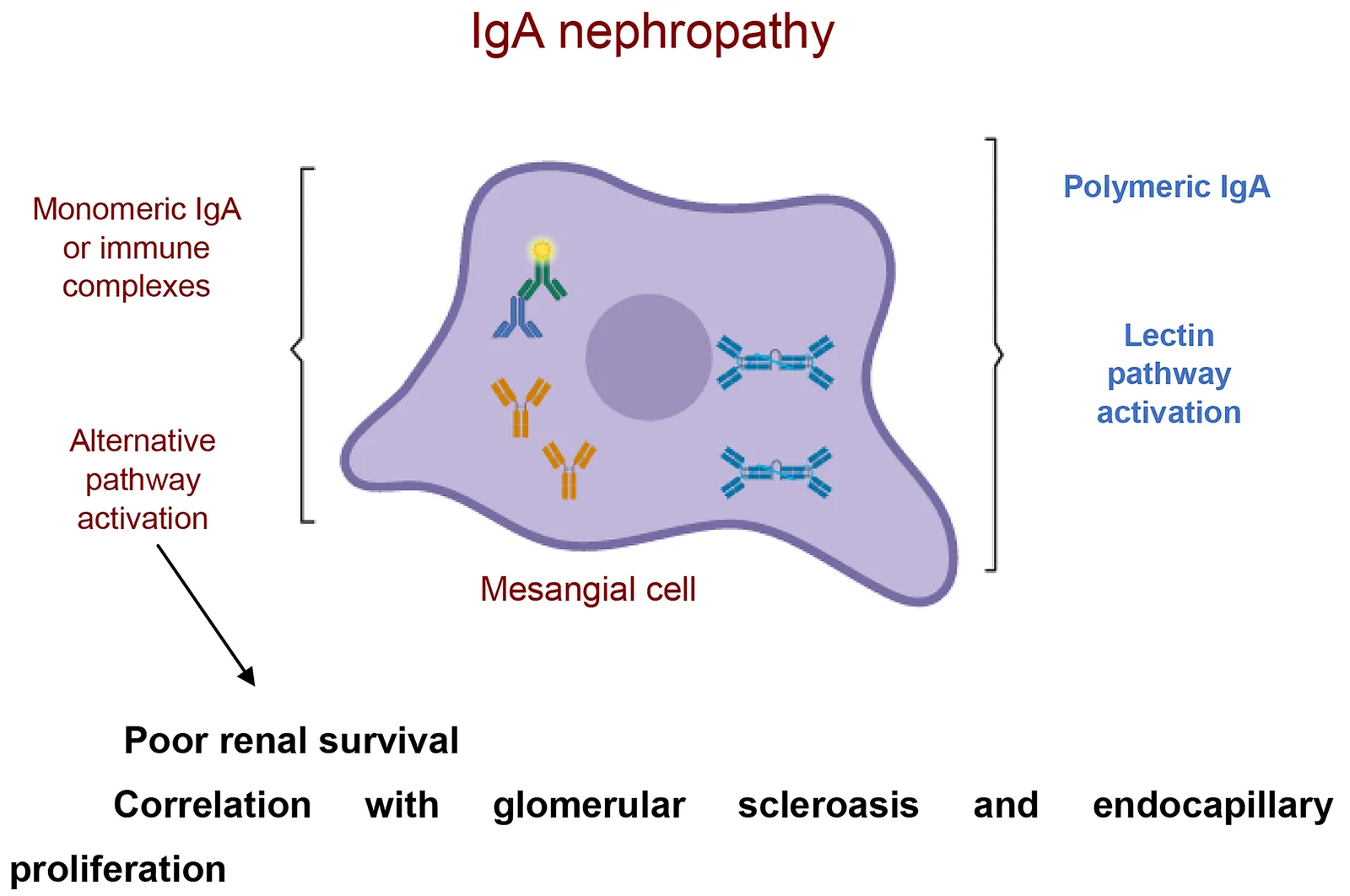
Complement system activation in IgA nephropathy. Ig: immunoglobulin.

There is still controversy regarding the treatment and prognostic scores for IgA nephropathy. Therefore, it is important to improve understanding of the role of the complement system in the pathogenesis of the disease, as well as to develop new tools for prognostic assessment and therapeutic decision-making.

## AAV and the complement system

One of the most common classes of vasculitis in patients over 50 years of age is the group of AAVs, all of which involve small vessels. ANCAs can target enzymes typically found in neutrophils, including myeloperoxidase (MPO) and proteinase 3 (PR3), referred to as MPO-ANCA and PR3-ANCA, respectively. These forms of AAV are characterized by focal, necrotizing lesions that create large gaps in the capillary wall, absence or scarcity of immunoglobulin deposition in the affected vessels, and a nephritic pattern. They can also cause extrarenal (systemic) symptoms. The main entities within the AAV group (37) are granulomatosis with polyangiitis (formerly Wegener's granulomatosis), eosinophilic granulomatosis with polyangiitis (formerly Churg-Strauss syndrome), and microscopic polyangiitis.

The characteristic lesion in the glomerulus and other vessels of the kidney and body consists of necrosis in the vessel wall, which releases plasma components, including coagulation and thrombogenic factors, thereby activating the coagulation cascade to produce fibrin. The resulting fibrin-filled necrotic area is known as fibrinoid necrosis (38).

There is ample evidence for the pathogenicity of ANCA. In 2002, Xiao et al. (39) provided the first convincing *in vivo* evidence for the pathogenicity of ANCA by transferring anti-MPO spleen cells from immunocompromised mice into other mice, inducing crescentic glomerulonephritis with proteinuria and reduced GFR. Other studies have implicated PR3 in the pathogenesis of granulomatosis (40).

Both PR3 and MPO enzymes can activate neutrophils, causing the release of free radicals, degranulation with lytic and proinflammatory enzymes, alternative complement pathway factors, and the release of neutrophil extracellular traps containing DNA with bound plasma proteins, which can cause tissue injury and activate the immune response. Xiao et al. (39) also demonstrated the importance of the alternative complement pathway in the pathogenesis of ANCA vasculitis. To investigate the roles of the three complement pathways in mediating anti-MPO-induced necrotizing and crescentic glomerulonephritis, they evaluated the induction of ANCA vasculitis by anti-MPO IgG in mice deficient in C4 or factor B. They observed that neither the classical nor the lectin pathway is required for the induction of this model of ANCA vasculitis. This finding supports the assertion that AAV is not a typical form of immune complex-mediated inflammation, which involves complement activation through the classical or alternative pathway. Therefore, the hypothesis was raised that neutrophil activation by IgG ANCA would cause the release of factors that activate the alternative complement pathway. Although no specific factor responsible for this activation has been identified, some candidates exist, such as properdin, which is synthesized by neutrophils and stored in granules released during activation (Figure 4). In addition, C5a stimulates the release of properdin from neutrophils, supporting the hypothesis that neutrophil activation occurs in an amplification cycle caused by C5a and properdin (1,21,41-43).

**Figure 4:**
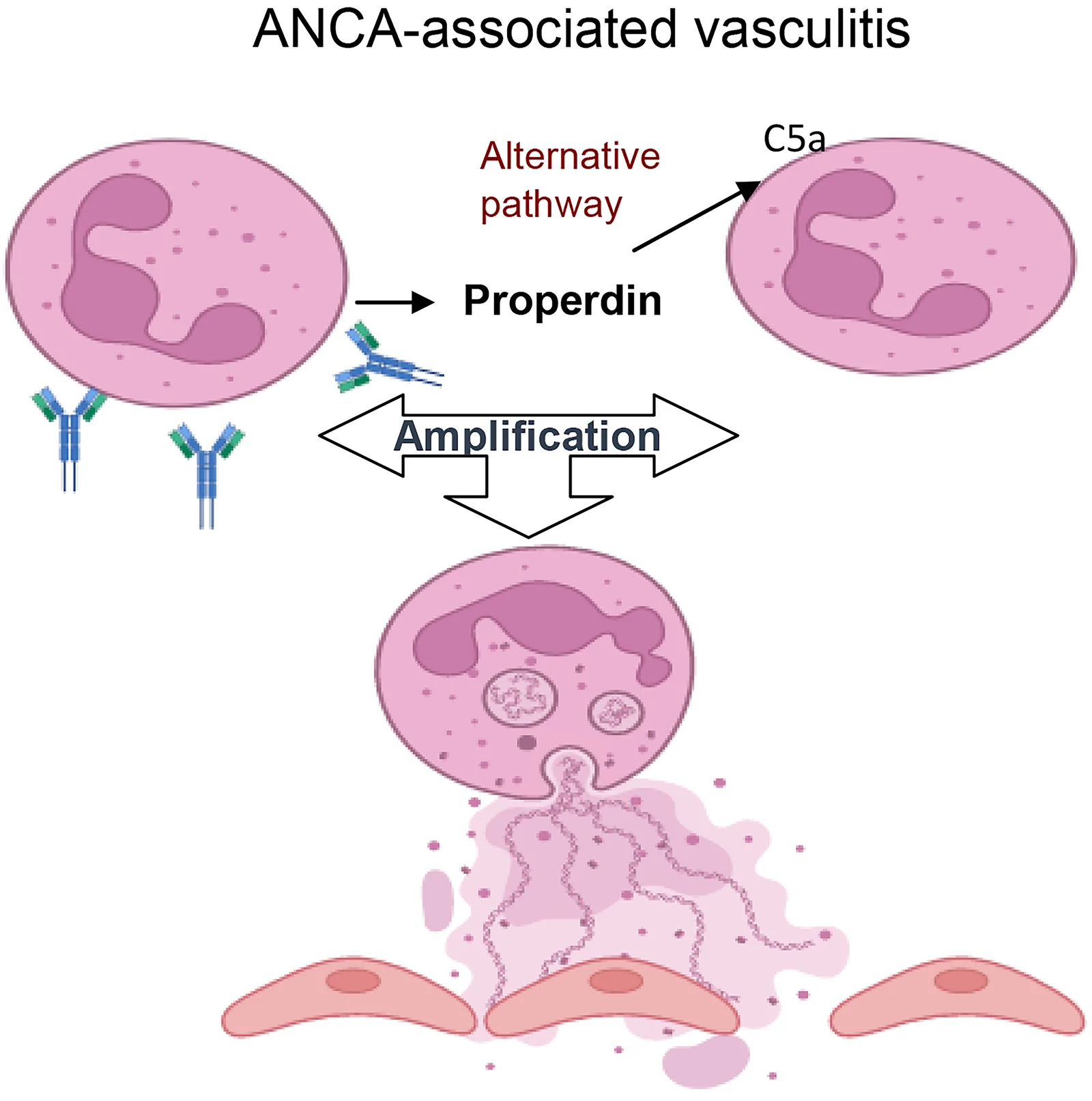
Antineutrophil cytoplasmic antibody (ANCA)-associated vasculitis and the complement system.

## Treatment with complement blockers

New studies have demonstrated the therapeutic potential of drugs that block the complement system, especially in IgA nephropathy and AAV. To date, treatment decisions in IgA nephropathy have been based on the degree of proteinuria, GFR, and tools that predict the risk of disease progression. In most cases, proteinuria greater than 0.5 g/24 h has been considered a factor associated with a higher risk of progression. Initial treatment mainly consists of antiproteinuric drugs, blood pressure control, and management of associated cardiovascular risk factors. The use of corticosteroids remains controversial because randomized clinical trials such as the Supportive versus Immunosuppressive Therapy Of Progressive IgA Nephropathy (STOP) trial (44) and the Therapeutic Evaluation of Steroids in IgA Nephropathy Global (TESTING) trial (45) have shown the non-superiority of corticosteroids and reported the side effects of corticosteroid therapy, respectively. The use of mycophenolate is reserved for specific populations, such as individuals of Chinese descent (46). However, recent studies have shown the significant involvement of the complement system, especially its alternative and lectin pathways, in the pathogenesis of this glomerular disease, leading to the investigation of new medications targeting various activation pathways of this system. Notable agents include narsoplimab, a MASP-2 inhibitor (47) that acts via the lectin pathway to form C3 convertase, and iptacopan (48), a potent inhibitor of the proximal complement pathway that binds to factor B and blocks the alternative complement pathway.

Preliminary data from ongoing studies have shown that two C5 inhibitors (ravulizumab and cemdisiran) and one C5a receptor antagonist (avacopan) provide a significant reduction in proteinuria. Therefore, in the latest (2024) Kidney Disease: Improving Global Outcomes clinical practice guideline for the management of AAV (49), based on the results of a phase 3 clinical trial, it is recommended that avacopan be used in the induction phase as a corticosteroid-sparing medication or for recurrent disease (50). The main anti-complement agents mentioned in this review, along with their targets and development status, are summarized in Table 2 (49-55).

**Table 2:** Complement-targeted therapies: selected clinical evidence and reported endpoints.

Glomerulopathy	Therapy	Target	Reported endpoints	Evidence and reference
IgAN	Iptacopan	Factor B	Proteinuria: 38%; Hematuria: NR; eGFR: NR	Phase 3; interim proteinuria reduction at 9 months 49
IgAN	Ravulizumab	C5	Proteinuria: 30%; Hematuria: NR; eGFR: NR	Phase 2 RCT with proteinuria reduction 51
IgAN	Narsoplimab	MASP-2	Proteinuria: NR; Hematuria: NR; eGFR: NR	Phase 3 program discontinued after interim analysis (52,53).
IgAN	Cemdisiran	C5 (RNAi)	Proteinuria: NR; Hematuria: NR; eGFR: NR	Phase 2 RCT; reported results in IgAN 54
AAV (GPA, MPA)	Avacopan	C5aR1 (CD88)	Proteinuria: NR; Hematuria: NR; eGFR: NR	Phase 3 in AAV (ADVOCATE) 50
Primary MN	Eculizumab	C5	Proteinuria: NR; Hematuria: NR; eGFR: NR	Clinical evidence inconsistent/limited in primary MN 55

IgAN: IgA nephropathy; AAV: ANCA-associated vasculitis; GPA: granulomatosis with polyangiitis; MPA: microscopic polyangiitis; NR: not reported; eGRF: estimated glomerular filtration rate; RCT: randomized clinical trial.

## Conclusions

Activation of the complement system, whether local or systemic, is responsible for glomerular damage in various glomerulopathies, including those with normal complement function. New serum and urinary markers may play an important role in monitoring these diseases and predicting increased risk of progression. Additionally, new drugs that inhibit specific pathways and molecules in the complement cascade, which have been studied with increasing frequency, have shown benefits in reducing proteinuria and disease progression - outcomes important for patients.

## Data Availability

All data generated or analyzed during this study are included in this published article.
